# From liability gaps to liability overlaps: shared responsibilities and fiduciary duties in AI and other complex technologies

**DOI:** 10.1007/s00146-024-02137-1

**Published:** 2025-01-11

**Authors:** Bart Custers, Henning Lahmann, Benjamyn I. Scott

**Affiliations:** 1 eLaw - the Center for Law and Digital Technologies, Leiden University, Leiden, The Netherlands; 2 International Institute of Air and Space Law, and eLaw - the Center for Law and Digital Technologies, Leiden University, Leiden, The Netherlands

**Keywords:** Artificial intelligence, Chilling effects, Complex technologies, Shared responsibility, Fiduciary duties, Liability, Legal certainty, Liability gap, Liability law, Moral responsibilities, Negligence

## Abstract

Complex technologies such as Artificial Intelligence (AI) can cause harm, raising the question of who is liable for the harm caused. Research has identified multiple liability gaps (i.e., unsatisfactory outcomes when applying existing liability rules) in legal frameworks. In this paper, the concepts of shared responsibilities and fiduciary duties are explored as avenues to address liability gaps. The development, deployment and use of complex technologies are not clearly distinguishable stages, as often suggested, but are processes of cooperation and co-creation. At the intersections of these stages, shared responsibilities and fiduciary duties of multiple actors can be observed. Although none of the actors have complete control or a complete overview, many actors have some control or influence, and, therefore, responsibilities based on fault, prevention or benefit. Shared responsibilities and fiduciary duties can turn liability gaps into liability overlaps. These concepts could be implemented in tort and contract law by amending existing law (e.g., by assuming that all stakeholders are liable unless they can prove they did not owe a duty of care) and by creating more room for partial liability reflecting partial responsibilities (e.g., a responsibility to signal or identify an issue without a corresponding responsibility to solve that issue). This approach better aligns legal liabilities with responsibilities, increases legal certainty, and increases cooperation and understanding between actors, improving the quality and safety of technologies. However, it may not solve all liability gaps, may have chilling effects on innovation, and may require further detailing through case law.

## Introduction

Digital Artificial Intelligence (AI) technologies, including automated decision-making tools, chatbots, and emotion recognition systems, can cause different types of harm, such as those stemming from bias and discrimination, privacy and security issues, and manipulating people into decisions (Citron and Solove 2022; Favaretto et al. 2019; Cofone and Robertson 2017; Wood 2021). Cyber-physical AI technologies, such as robots, unmanned aircraft systems (drones), and self-driving vehicles can also cause physical harm, for instance, in case of a collision (Zhu et al. 2021). AI-based decision-support systems (DSS) for military commanders or lethal autonomous weapon systems (LAWS) deployed in situations of armed conflict can cause unlawful harm by targeting civilians in violation of the principle of distinction or by engaging lawful military objectives while incidentally injuring or killing civilians or civilian objects in violation of the principle of proportionality (Zhang 2024). When harm occurs, the question arises who is liable for the harm caused. In such situations, stakeholders may start apportioning blame to each other. Engineers and technicians regularly suggest that they only build the technology, and that it is for others to decide how and when to use it, whereas users often suggest that they only use technologies for the purpose for which these were intended or designed. In the case of weapon technology, for instance, manufacturers usually claim end-user responsibility; military commanders deploying the weapon might contend that the harm occurred due to the system limitations or malfunctioning, for which the manufacturer is responsible; victims of the technology, finally, might argue that their harm would not have occurred if the weapons were not manufactured in the first place or that the harm can be traced back to misuse by the military forces.

The starting point for legal liability is typically some form of responsibility.^1^ Responsibility can be established if an actor has some form of control or influence over a decision or action. The most common type of liability is liability through fault or intent, which means the person or entity that causes damage is liable if there is a duty of care and there is a breach of that duty. Via mechanisms of strict liability, any liability can be extended, for instance, via vicarious liability (e.g., parents are liable for their children, dog owners for their dogs, and companies for their employees and products)^2^ or product liability^3^ (manufacturers are responsible for the quality and safety of products they put on the market under both contract and tort law) (Wendehorst 2020; Glavaničová and Pascucci 2022). Legal liability can be grounded on who causes damage or who creates risks, but also on who benefits or who can prevent risks. All mechanisms assume some degree of control of an actor over a situation (cf. Conradie 2023).

If legal liability is not well-aligned with responsibilities, this may yield unsatisfactory results. These are referred to as liability gaps: situations in which liability is allocated in ways that do not align with (moral) responsibilities.^4^ In other words: applying existing liability rules leads to (morally) unsatisfactory outcomes. In this paper, four major types of liability gaps are distinguished.

1. The first type is liability gaps in which no one can be held liable (e.g., when technology takes over decision-making processes)
2. The second type is liability gaps in which the wrong actor is held liable (e.g., when the most proximate human operator is blamed for this circumstance alone)
3. The third is liability gaps in which more than one actor is held liable (and all eventually escape liability by apportioning blame to each other)
4. The fourth is liability gaps in which multiple legal regimes compete (and all actors escape liability by focusing on a legal regime that favors them).

All types can typically occur in the context of autonomous, self-learning technologies such as those that utilize AI systems. If the technology makes autonomous decisions, there may be no human actor with fault or intent. If the technology causes damage while it functions as intended (for instance, the optimal decision for an autonomous drone may be to autoland, rather than collide with a passenger aircraft), product liability may be hard to apply because the product did exactly what it was supposed to do (which is limiting unavoidable damage). In the absence of vicarious liability, this may yield the unsatisfying outcome that no one is liable (type 1), that the liability is shifted from the technology to the most proximate human operator, establishing liability to the wrong actor (type 2), more than one person is held liable (type 3) or multiple legal regimes apply (type 4) (Elish 2019). Research has identified multiple such liability gaps in legal frameworks addressing liability (De Conca 2022).

There are also practical issues that contribute to liability gaps. Liability may be hard to establish because of the many stakeholders involved in complex technologies, which constitute a non-transparent landscape of actors and their roles, the so-called problem of many hands (Thompson 1980). This may be the case from the perspective of users, but also from the perspective of companies that may lack an overview of who contributed to components delivered to them via complex supply chains (incl. bespoke products and off-the-shelf components). Liability may also be hard to attribute in practice because of power asymmetries between users and technology companies or among technology companies (cf. Elish 2019).

To address these liability gaps, the legal affairs committee of the European Parliament has suggested that AI and robots should perhaps have legal personhood in the future (Hern 2017). Such legal personhood for AI and robots would involve rights and duties for them, but it is unclear which rights and duties these would be and how that would work. Due to these complexities, this proposal never made it to the agenda of the European Commission. In this paper, therefore, a different approach is taken. To avoid liability gaps and the unsatisfactory results that come along with them, this paper explores the concepts of shared responsibilities and fiduciary duties, in which overlap in responsibilities could serve as a basis for legal liability and avoid these gaps. The concept of shared responsibilities is based on the observation that the development, deployment, and use of complex technologies are not clearly distinguishable stages, as often suggested, but processes of cooperation and co-creation (Fuglslang 2001). At the intersections of these stages, shared responsibilities can be observed rather than gaps in responsibilities. Shared responsibility means that multiple stakeholders are proportionally responsible for a particular action or outcome. This is not the same as collective or joint responsibility (List and Pettit 2011; Björnsson 2020), in which all actors are responsible, regardless of fault or intent, but zooms in on the responsibilities each actor has in these processes. None of the actors have complete control or a complete overview and, as a result, all actors are restricted in their control and influence. Nevertheless, many actors have some control or influence, creating responsibilities based on fault, prevention, or benefit. Typically, an important distinction relating to the division of responsibilities is between diagnosis of and response to potential risks (Vedder and Custers 2009). An actor may not have a solution to a specific problem that is identified, but may have a responsibility to signal this to other actors. This is where the concept of fiduciary duties can be helpful. A fiduciary duty involves actions taken in the best interest of another person or entity. This can include a duty of care, loyalty, good faith, and prudence. In the context of AI and other complex technologies, it involves taking into consideration other actors and their vulnerabilities into one’s decisions, actions, and behavior.^5^

This paper does not focus on a specific jurisdiction and the existing legal rules for liability. Instead of taking a black-letter law approach, it explores what future frameworks for liability could look like. In that sense, a moral perspective is taken, rather than a legal perspective. This moral perspective means the focus is on how responsibilities and liabilities *should be* assigned or distributed to achieve satisfactory outcomes, rather than how they must be assigned based on existing legal rules. In this paper, the term (legal) *liability* is used for (backwards looking) legal liability in existing legal frameworks and the term (moral) *responsibility* is used for the (forward-looking) possible approaches (cf. Poel et al. 2015).

This paper is structured as follows. Section 2 examines basic liability mechanisms and the liability gaps that may occur when dealing with complex, self-learning technologies like AI. Section 3 investigates how a different approach, focusing more on shared responsibilities and fiduciary duties, could address liability gaps and discusses the pros and cons of this new paradigm. Section 4 wraps up with conclusions and recommendations.

## Liability law and liability gaps

### Liability mechanisms

As a general principle, if someone causes harm, that person is liable for it, because of fault or negligence. Exceptions may apply depending on the situation, for instance, on the level of control and knowledge that a person has. Typically, children or people with limited cognitive capacities can be exempted from this legal liability because from a moral position, they should not be held responsible to the full extent. To avoid situations in which no one would be liable for harm or damage caused by these groups, they are usually covered by mechanisms of vicarious liability. This is an extended liability for parents for their children, owners for their animals, and employers for their employees. Such vicarious liability does not require any fault or negligence of the parent, owner or employer: it is grounded on the (generally accepted) responsibility that parents have over their children (and owners over their animals and employers over their employees). Parents should avoid that their children cause harm or damage, and if despite all efforts of parents, children nonetheless cause harm or damage, the parents are liable for it.

Looking more closely at how responsibility and liability are attributed to actors, there are three underlying mechanisms that can be used for this. In each of these mechanisms, there is an emphasis on the level of control or influence that an actor has over an event. Before discussing these three mechanisms, it is important to note that in complex technologies, the various actors involved may have very different expertise, abilities, control, and influence on the development and use of these technologies. None of the actors have complete control or a complete overview of the situation. Actors in the early stages of research and development may not be able to oversee all risks and benefits of a technology and actors in the final stages of deploying and using a technology are unable to influence choices in design and functionality. As a result, all actors are restricted in their control and influence.

The first mechanism is attributing responsibility and liability on the basis of fault or negligence, i.e., on what an actor did or omitted to do. If someone breaches a duty of care and causes harm, that actor is liable. The actor controlled their own actions and perhaps should not have acted as they did. In the case of omissions, if there was an obligation to act^6^ but the actor did not act to prevent any harm from being done, then the actor could be held liable. There is some (assumed) control, often in the form of intent, in these actions or omissions to act. This control or intent does not always have to be very explicit. Legally, it can sometimes be construed as ‘accepting the consequences of acting or not acting’ or ‘level of care’ that could reasonably be expected, rather than knowing the actual consequences of the behavior (Zech 2021; Wagner 2019). Slightly different, but still in this category of fault or negligence is responsibility based on causing danger, with subsequent liability. Although causing danger (which may never materialize) is not the same as causing actual harm, creating danger (or a higher level of danger) can be considered as doing harm in itself (Solove and Citron 2017).

The second mechanism for attributing responsibility and liability is strict liability, which is essentially based on considerations of prevention. Prevention does not refer to negligence or omissions to act, but to taking active measures to avoid harm or damage, including avoiding or mitigating increased levels of danger. This prevention seeks to mitigate incidental harm related to actions. It requires a balancing act between several interests. Actors are responsible for considering different perspectives, and the harms and damages they may cause from these perspectives. A typical example of prevention is when a manufacturer does not build any safety measures into a technology. If the technology then causes any harm, the manufacturer is responsible and can be held liable, as it is an actor (sometimes the only actor) that could have prevented the harm. This is different from negligence, as the manufacturer is not expected *not* to produce the technology.^7^ Instead, it may be reasonable to expect the manufacturer to take safety measures into account. Most forms of product liability are also based on this mechanism: even though users may use products in harmful ways, manufacturers have some responsibility to prevent such unsafe use (Geistfeld 2021). Responsibility and liability through prevention can be easier to understand through proof by contradiction: for instance, if no one were responsible and liable for actions by children, not attributing liability would create unsatisfactory results when they cause harm. This is because some degree of control over children by their parents can be reasonably assumed. Similarly, the mere existence of product liability laws is likely to encourage companies that technology put on the market is safe.

The third mechanism for attributing responsibility and liability is on the basis of benefits. The moral reasoning of beneficiary responsibility is that those who benefit from particular actions or situations should bear some level of responsibility (Young 2006; Chung et al. 2021). For instance, if a company benefits from exploiting natural resources, it may be argued that it has a responsibility to ensure responsible and sustainable use of these resources and to mitigate any negative effects on the environment or local communities. Such responsibility can be derived from a supposed duty of care or from control and influence. Those who have more influence may bear a greater responsibility for the situation, particularly if they have the ability to shape the situation. Responsibility on the basis of who benefits is often not codified in legal liability. Arguably, some forms of strict liability, including product liability, are based at least partially on this mechanism. A typical example here is the precautionary principle enshrined in EU environmental law, in Article 191(2) TFEU. This article states that EU policy on the environment shall be based on the precautionary principle. This principle states that if an activity (e.g., a new technology) may lead to harm, actions should be taken to avoid or mitigate that harm. In the Artedogan case, the EU Court of Justice ruled that the precautionary principle extends beyond environmental law (CJEU 2006). Hence, although this third mechanism is less prevalent in current legal frameworks, it is becoming increasingly relevant in the context of complex technologies like AI. For highly autonomous technology, it may become very hard to use prevention as a basis for allocating responsibility and liability, simply because actors developing, deploying, and using the technology may be unable to prevent certain harms, either because they may not foresee certain scenarios or because the technology acts autonomously. In these situations, attributing responsibility and liability on the basis of benefits may offer an alternative approach.

All three mechanisms are based on some form of control or influence that actors have. However, in many situations in which complex technologies play a role, actors may have limited control over the behavior of an automated or autonomous system. In such situations, these mechanisms may not function properly and responsibility for an action may be misattributed to a human actor who has limited control, which can lead to liability gaps (see Sect. 3).

### Liability in complex technologies

**Fig. 1:**
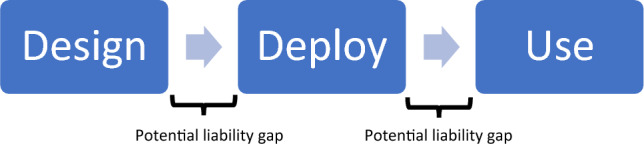
Simplified model of the stages of the process of putting technologies on the market. Actors are typically related to one stage, potentially creating liability gaps between stages

The process of putting technologies on the market typically consists of a multitude of stakeholders. One of the most simplified models consists of three actors: the first designing, developing, and manufacturing the technology, the second deploying the technology, and the third using the technology (see Fig. 1). A typical example to illustrate this are aircraft. Commercial aircraft are designed and manufactured by large aircraft manufacturers like Boeing and Airbus. They are then sold to airlines that provide air transport services to passengers or cargo. These airlines deploy the technology. Customers and freight forwarders use the technology when booking airline tickets or shipping freight. In the context of military technologies, large defense contractors such as BAE Systems produce the systems, whereas states can be said to be the deployers. The users of the systems are the commanders and other personnel who field them for individual missions.

For some technologies, it can be the same actor that designs, deploys, and/or uses the technology. For instance, when a large company builds its business intelligence system for inventory management or customer relations, the designer, deployer, and user are the same. In many cases, however, these are different entities. In fact, when zooming in on each of these three stages, in practice there can be many more actors in each stage.

In the first stage of design and manufacturing, complex technologies are typically assembled using many semi-finished products. For instance, when building an aircraft, all kinds of components (such as engines, dashboards, windows and chairs) can be delivered by suppliers and integrated into the final product when assembling the aircraft. In fact, manufacturers of components like engines (e.g., Rolls Royce, Pratt & Whitney and GE) may have their own suppliers of semi-finished products and components. As such, there can be many more actors than only the large aircraft manufacturers that put their name on the final product. In addition to outsourcing the manufacturing of semi-finished products, each of these companies can also outsource other processes, such as the research (e.g., aerodynamics, energy use and crash tests), the design (e.g., appearance/look-and-feel, aerodynamics and user-friendliness), or development (e.g., testing and evaluation), and sourcing the raw materials. This supply chain, as well as the final assembly, may span several countries.

In the second stage, there can be many actors deploying the technology. In the case of commercial air transport, airlines buy or (wet or dry) lease the aircraft. Airlines may use the aircraft to transport passengers, cargo, and mail. They rely on intermediaries, such as logistics companies, travel and booking agencies, and ground handling companies. Insurance companies will assist in covering any accidents and incidents during transport. Manufacturers may also use aircraft, such as Airbus which has an Air Operator Certificate for its Beluga aircraft. These aircraft are used to transport resources between the different assembly points.

In the third stage, there are different types of customers and consumers that make use of the services offered by those deploying the technology. In the case of aircraft, this can be both in the types of actors and in the number of actors. The types of actors can be, for instance, individuals booking airline tickets or companies booking freight. Since billions of people use aircraft, the number of actors can be considerable.

When looking at liability in case of any incidents or accidents, the focus is usually on individual entities that can be legitimate bearers of (moral) responsibility and legal liability. Most legal frameworks make use of concepts like product liability, in which companies are liable for their products and services, and vicarious liability, in which companies are liable for their employees. This means that when aircraft manufacturers make use of semi-finished products, from the perspective of deployers and users, they are responsible for (the well-functioning of) the entire aircraft. If, for instance, an accident happens due to the malfunctioning of an engine supplied by a subcontractor, the airline company deploying the aircraft can hold the aircraft manufacturer liable (e.g., under warranty). It is not required to find out who delivered the malfunctioning engine. The aircraft manufacturer can, in turn, hold the supplier of the engine liable. Similarly, if an employee of the aircraft manufacturer drafted a flawed or dangerous design, the company may be liable for this. Only internally, they can decide to take further action toward the employee. For these reasons, actors in research, design, development, manufacturing, and assembling technology are aggregated in Fig. 1. Aircraft undergo a strict certification process, whereby Design Organizations Approvals and Production Organizations Approvals are granted to manufacturers by competent authorities. Further, the aircraft type must have received a Type Certificate, and the individual aircraft must have a certificate of airworthiness. Again, to gain these, there is a lot of scrutiny by the competent authorities (e.g., civil aviation authorities) and the assessment is based on law. Therefore, if the manufacturer follows the rules, the question can arise whether it can still be held liable for a damage or that perhaps the competent authority is responsible. This adds another layer of complexity as the competent authority is often a public body.

Actors that deploy technologies are aggregated in Fig. 1. Under most legal frameworks for consumer protection law, consumers have the right to remedy or redress against the actor from whom they purchased something or have a contract with.^8^ In the case of airlines, when a consumer buys an airline ticket at a travel agency, this agency serves as the point of contact and, from the consumer’s perspective, so it would seem to follow that it is liable in case something goes wrong. If the issue is caused by the airline, the passenger instead has a direct cause of action against the airline under the Montreal Convention 1999 or Warsaw Convention 1929. Because of this, actors deploying technology are aggregated with intermediaries, resellers, and sales offices in Fig. 1.

In essence, Fig. 1 shows the aggregation of legal liability for technologies. Within each stage, the liability of many actors is concentrated on one major actor who can hold subcontractors liable where needed. However, Fig. 1 also shows that there may be liability gaps between the major/aggregated actors. In the case of incidents or accidents, a designer or manufacturer of a technology may claim that there was an incorrect use of the technology, attempting to put liability on those who deploy or use the technology.^9^ Usually, it then needs to be investigated what caused the damage: a flawed product or wrong use.^10^ However, in the case of complex technologies, both may be complicated to establish. Products are flawed when they do not perform as may have been expected, but it may be unclear what could be expected. Wrong usage occurs when technology is used in other ways than its design and functionality are intended for, but for complex technologies, the scope of functionality and applications may be unclear, as contexts of deployment may vary widely. As a result, liability may be allocated to the wrong actor or no actor, creating liability gaps, as will be discussed next.

### Liability gaps

As shown in Fig. 1, where liability is not aggregated to specific actors, liability gaps may open up, at least in the case the applicable legal framework does not explicitly provide for such situations by assigning liability to one specific class of actors by default—as for example under the Montreal Convention in the context of civil aviation.^11^ If such a legal construction is missing, actors may start pointing at each other to avoid liability and it can become unclear who is liable since it is unclear what could be expected from the technology and/or how the technology should be deployed and used. Liability law tends to focus on putting the blame on one actor, whereas, in complex technologies, several actors may have responsibility. Furthermore, questions of jurisdiction and *forum non conveniens* can arise. In international disputes, more than one court may have jurisdiction. In such cases, actors may be able to escape liability by hiding behind jurisdictions or legal regimes that are most favorable to them.

These issues can be aggravated when technologies are used that can act autonomously and that are self-learning. Autonomous technologies can make decisions that are not pre-determined. For instance, autonomous drones can be designed to make evasive maneuvers to avoid collisions with buildings, other aircraft or birds (Gugan and Haque 2023). The exact movements are not programmed, the drone is equipped with software that searches for the optimal solution given the circumstances. Depending on the situation, the drone can decide to move underneath or above another flying object, or follow another course of action, like slowing down, changing direction or landing.

Self-learning technologies, such as technologies equipped with AI, can show even less predictable behavior (Yampolskiy 2020). Most technologies generate reproducible output, where the same input always results in the same output. This is not the case for self-learning technologies: the exact same input may result in different outputs at a later time because the technology has learned to assess the input differently. If technology is self-learning, even those who designed and built the technology may at some point be unable to explain how the technology works or predict how it will react. In case of incidents or accidents, it may also be hard to reverse engineer how the technology came to a particular behavior or a particular decision (i.e., an AI black box).^12^ Even when it is possible to reverse engineer a particular decision, the understanding of the technology is just a snapshot. After some time, the technology will have evolved, and the understanding will get outdated.

AI tools are self-learning, which complicates the application of the existing liability rules. A typical example is a self-learning autonomous drone.^13^ Suppose this drone needs to avoid collision with a commercial aircraft carrying passengers and crashes on the ground, wounding a person who happens to be there. It could be argued that this person should be compensated for the inflicted harm and, therefore, be able to hold someone liable. However, it may be hard for the victim to hold the operator of the drone liable, as they could not influence the behavior of the drone in any way. It may also be hard to hold the seller or manufacturer of the drone liable, as their product did what it was supposed to do, i.e., avoiding a collision with an aircraft, which could have caused a major accident. Similarly, the idea behind AI-supported weapon systems is that they are typically programmed to autonomously identify and engage targets based on machine learning algorithms that are trained with large sets of labeled and unlabeled data of lawful and unlawful potential targets. For this reason, the ability to reliably predict how the self-learning system will behave during actual deployment is by default extremely limited (Custers 2003). If such a system harms a civilian or civilian object in a way that constitutes a violation of applicable international humanitarian law, it may be difficult to hold the military commander who deployed the system liable (Zhang 2024). This is at least the case if there was no person ‘in the loop’ and the system was otherwise deployed within the limits of the law. Therefore, such situations can result in a ‘liability gap’, in which liability cannot be allocated in a satisfying way (De Conca 2022; Bertolini 2013).

In the literature, there are different definitions of what a liability gap means.^14^ Liability gaps are often conceptualized as situations in which nobody is liable for damage or harm that has occurred (Kwik 2023; Munch et al. 2023; Himmelreich 2019). However, from the perspective of liability law, there is nothing wrong with this in principle and it certainly does not mean there is a gap.^15^ For instance, if a person is struck by lightning, there is harm, but no one can be held liable for this if there is not a duty of care.^16^ As a result, the harm will remain on the victim without any compensation, although the victim is not responsible or liable. Victims may also have a role as they may have contributed to the injury, such as acting in a reckless way. The fact that liability cannot be assigned to anybody does not mean there is a liability gap: the law adequately addresses this situation, as most people would agree that from a normative perspective, no one is responsible or liable.^17^

Liability gaps refer to situations in which liability cannot be allocated in a satisfactory way. In other words, the way in which legal liability is allocated does not align with (moral) responsibilities (Matthias 2004; Danaher 2016). This makes liability gaps morally problematic by definition (Danaher 2022; Kneer and Christen 2023). In the example of an autonomous drone that crashes into a person, it may feel morally wrong if the victim is not compensated for the harm. There is a difference between being hit by lightning, caused by a natural phenomenon, and being hit by a drone, caused by human actions. Actors that deploy and use drones consciously and knowingly choose to activate the technology and that may bring along some responsibility. They may create (an elevated level of) danger with this. If something goes wrong but the law does not assign any liability related to this responsibility to these actors, this creates a liability gap.^18^

In this paper, the latter meaning of liability gaps (i.e., non-alignment) rather than the former (i.e., lack of liability) will be used. However, both meanings are closely related. A liability gap in the sense of non-alignment can occur in situations in which no actor can be held liable or in situations in which the wrong actor is held liable. If no actor can be held liable, both meanings can converge if the outcome is unsatisfactory. If the wrong actor is held liable, there would not be a liability gap in the sense of lack of liability. The liability gaps in Fig. 1 at first sight may look more like liability gaps in which no one can be held liable but should be regarded as liability gaps in which either no actor or the wrong actor is held liable despite responsibility.

Four types of liability gaps must be distinguished (see Sect. 1). The first type is liability gaps in which no one can be held liable. This typically occurs when technology takes over decision-making processes, like in the example of autonomous drones. If there is no human in the loop, no one may be liable, which is likely to yield unsatisfactory outcomes. The second type is liability gaps in which the wrong actor is held liable. In case the technology is not fully autonomous and there are humans in the loop, any actions or decisions of the technology could be blamed on the most proximate human operator. In other words, the liability mechanisms described in Sect. 2.1 may not function properly and responsibility for an action may be misattributed to a human actor who has limited control. This is sometimes referred to as moral crumple zones, in which responsibility is attributed to the most proximate human operator, to protect the integrity of the technological system (Elish 2019). The third type is liability gaps in which more than one actor is held liable. The risk here is that all actors eventually escape liability by apportioning blame to each other, or that liability is not distributed proportionally, or both. Weapons technology is an example here, as manufacturers may push responsibility for any victims to users, whereas users may push responsibility for any victims toward manufacturers. For victims, it may then be hard to hold any of them liable, let alone both and in a proportionate way. The fourth type is liability gaps in which multiple legal regimes compete. Also, here there is the risk that all actors escape liability by focusing on a legal regime that favors them. Developers and manufacturers of complex technologies can argue they are in compliance with all legal requirements in the jurisdiction in which they operate (and therefore not liable), but when these technologies are deployed in other jurisdictions (in which they are liable) it may be impossible to sue them.^19^

## Toward shared responsibilities and fiduciary duties

### The concept of shared responsibilities

In practice, the development, deployment, and use of complex technologies are not always clearly distinguishable stages, at least not at their intersections (Fuglslang 2001). What is sometimes understood as separate stages in practice is often an iterative approach or circular life cycle^20^ in which new technologies and new applications of existing technologies are built on each other (La Fors et al. 2019). In these processes, the different stages may sometimes overlap or run in parallel, the cooperation can be fluid and control can be shared. Joint cooperation can lead to decisions and actions that cannot be traced to individuals or single entities (List and Pettit 2011; Björnsson 2020).^21^ Sometimes the stages in the process are even non-sequential. Complex technologies are never really finished but are continuously updated and revised, also when they are already used in practice. This is particularly the case for self-learning technologies such as AI. AI systems can develop over time and improve their performance after they have been fed with large amounts of data.^22^ That can be training in laboratories, but usually it also (particularly) involves training of the systems in practice. In situ learning can significantly contribute to the performance of these systems (McFarland and Assaad 2023). For instance, personal assistants using voice recognition will train themselves on the basis of user input. The large amounts of data that become available when training complex technologies in practice is a major reason for some companies (like ChatGPT) to put their products on the market for free. All user input contributes to further training of the models. Emergent behavior, i.e., behavior that appears after the implementation of a technology, can only be addressed after this (Serugendo et al. 2006).

This means that those who deploy and use complex technologies are actively involved in the design and the further improvement of these technologies.^23^ It could be argued that along with this involvement comes some level of responsibility. If technology developers involve users in their processes, they show greater responsibility. Users, in turn, have a responsibility to contribute to this in adequate ways, for instance, by providing correct data; even if this responsibility is not necessarily a legal obligation or legally enforceable.^24^ It could be argued that those who deploy and use these complex technologies are essentially using unfinished products, which means that some responsibility for the deployment and use should be transferred back or shared with those who design and manufacture these technologies. In other words, if the stages for putting technologies on the market are not clearly distinguishable, neither can the responsibilities be clearly distinguished. When multiple actors are co-creating these technologies, all of them may have some (smaller or larger) share of responsibility. This also involves actors that do not make final decisions, such as advisory, testing or facilitating staff, as decisions and actions are rarely free of influence from others (Levy 2018).

Liability law generally does not reflect this practice in at least two ways. First, in practice, many actors may have contributed to a larger or smaller extent to an event in which harm or damage could occur. Liability law focuses on whether an actor can be held liable but is less equipped to identify the smaller bits and pieces of responsibility across multiple actors. Second, liability law can only deal with legal entities. In practice, however, many actors transfer some level of control to complex technologies. The cooperation between actors and technology, and among actors can be fluid and control can be shared. The dynamics of shared control can be very complicated, leading to issues regarding allocating responsibilities (Jones 2015). Transferring control to complex technologies may not always imply the transfer of liability, but if an actor no longer has control, liability may be difficult to establish. For example, if an autonomous weapon system makes the decision to engage a civilian target under violation of international humanitarian law without any human operator being in the loop, based on data input acquired during the deployment phase, it may be impossible to hold a legal entity liable for the harm caused, such as a member of the armed forces of the deploying state (Zhang 2024). Some argue that if the technology has full control, the technology itself (e.g., the autonomous weapon system) is responsible and, therefore, should be liable (Hern 2017; Daily Wire 2018; for criminal law, see also Gless and Weigend 2014; Gless et al. 2016). However, AI systems and other complex technologies are not legal entities and cannot be held liable under current legal frameworks. It is also unclear what such liability could even look like in practice.

**Fig. 2:**
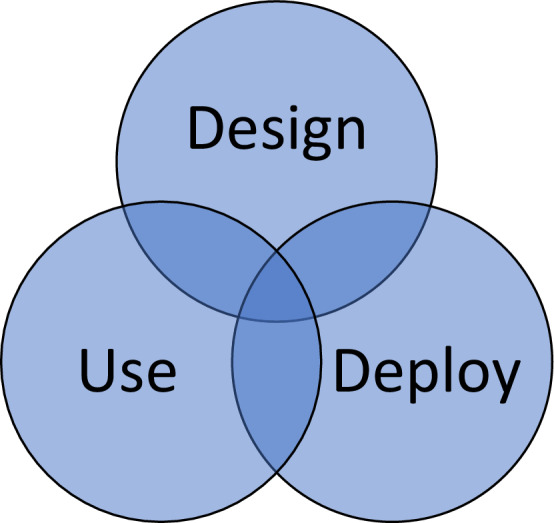
Shared responsibilities in the stages of the process of putting technologies on the market

Figure 2 shows an adjusted perspective on the responsibilities and liabilities of actors, with three major modifications compared to Fig. 1. First, with the circular positioning, Fig. 2 better reflects the non-linear approach to putting technologies in the market. Second, by removing the arrows, it becomes clearer that putting these complex technologies on the market often is an iterative process, in which development can go back and forth between stages. Third, the overlapping circles show the shared responsibility. At the intersections of the stages, there often is interaction between actors and, therefore, potentially also shared responsibilities may exist. The shared responsibility then exists on the basis of the involvement of multiple actors.^25^

Figure 2 better reflects the processes of co-creation and cooperation than Fig. 1. It also better recognizes that the steps should not be a cascade in which all residual liability ends at the user. However, it makes things much more complicated, as it becomes clear that different actors and their behavior are much more intertwined than it may appear at first sight. Dealing with liability overlaps is much more intricate than dealing with liability gaps, both from a moral and legal perspective. Identifying the boundaries of actors within a system of shared control can be tricky (Elish 2019).

From a moral perspective, responsibilities in the overlapping stages can be allocated based on fault or negligence. If manufacturers put early designs of a technology on the market for in situ testing and further development, these versions may still have issues and can potentially cause more damage than final versions. On the one hand, it is responsible behavior to involve users in the early stages; on the other, it may be irresponsible to expose users to unsafe and untested technologies. Putting all responsibility on the stage of research and development may be complicated simply because not all possible risks and benefits can be known at that stage. Putting all responsibility on users is not realistic as users will lack knowledge about what they can reasonably expect from the technology. Assessing the levels of responsibility of different actors can quickly become a quagmire.

Shared responsibility does not mean that actors cooperating have the same responsibility or liability or that they are equally responsible or liable (like in joint and several liability in which each actor is individually responsible for an entire obligation or damages, irrespective of proportion or fault). It means that they are in this together and that their level of responsibility and their share of liability has to be assessed on a case-by-case basis. Their shared responsibility then translates into a proportional liability, in which liability can be divided proportionally among the actors involved. It does not mean that they are all liable if something goes wrong, they will still only be liable to the extent they had some form of control over an event.

All three mechanisms explained in Sect. 2.1 can be used for assessing the level of control of actors and any responsibility that comes along with it. Fault and negligence may help to assess the existence, nature and size of any responsibility and liability of stakeholders working together. In fact, if some actors are not involved in a particular stage, this could also be considered an omission or negligence, for instance, when a manufacturer does not include users in any technology testing, or when users provide no feedback in case they are under an obligation to do so, for example, based on a contractual arrangement.

In some situations, however, prevention and benefit may be more suitable mechanisms to assess this. Take again the example of the drone that makes evasive maneuvers to avoid a collision but crashes. There is no fault or negligence, but there is a liability gap with unsatisfactory results regarding the allocation of liability. A mechanism based on prevention or benefit would allow for the reasoning that actors who could have prevented this outcome or actors who benefit from the deployment of the drone have a responsibility. When looking at prevention, actors have a shared responsibility to examine different scenarios and emergent behavior of the drone. It would be too easy to say that only the manufacturer is responsible and should have thought about all scenarios, especially if autonomous behavior is the very purpose of the technology, as with certain types of AI-based weapon systems or military DSS. The actors deploying and using the system should also have taken such eventualities into consideration and provided contingency scenarios. Hence, there is shared responsibility based on prevention: all actors could have considered and raised further measures to prevent this.^26^ Ubiquitous use of AI technology would require that our society further consider the risks involved.

Shared responsibility based on benefits can also be constructed in this case. All actors (apparently) benefit from the drone use. On that basis, they have a shared responsibility in case something goes wrong. Without these envisaged benefits, the actors would not have developed, deployed or used the drone, so they all have some responsibility in enabling a situation or facilitating a course of events. This kind of faultless responsibility does not focus so much on distributing reward and punishment, but rather on establishing feedback mechanisms that incentivize actors to improve outcomes (Floridi 2016; see also Taddeo et al. 2021).

Given that no actor has complete control or a full overview of the process, it makes sense to further distinguish types of control and influence. An important distinction relating to the division of responsibilities is between diagnosis of and response to potential risks (Vedder and Custers 2009). Many actors in the different stages have only limited insight into the risks and many have restricted means to respond. These restrictions particularly apply to other stages in which an actor is not involved. Typically, engineers and technicians in the research and development stages have limited influence on the introduction of new technologies into the market. Actors deploying and using the technology may influence how new technologies are introduced into society and how they are actually used. However, users usually have limited influence on the research, development, and production of these complex technologies. A distinction between responsibilities to identify risks and responding to risks can help determine (the level of) responsibility of an actor. Even if an actor cannot solve or mitigate an identified risk, there exists a responsibility to notify that risk, so that others are enabled to respond to the risk. For instance, if an engineer identifies an environmental risk in the production of a particular technology, it does not mean that the solution for this problem is this person’s responsibility. The responsibility is limited to signaling the risk.

The most practical way of translating any shared responsibility into legal liability may be introducing a reversal of the burden of proof in existing legal frameworks. All stakeholders are responsible (and therefore liable) unless they can prove they had no form of control whatsoever. Only showing they did not act would be insufficient to escape liability if they had some form of control. At the same time, the law should move away from the dichotomous approach that currently exists in liability law. Instead of a legal framework that is focused on determining whether an actor is legally liable or not, the focus should be on the question of to what extent an actor was able to control or influence a situation or course of events leading up to an incident or accident. In other words, liability should not be an all-or-nothing question, but a qualified assessment of roles and responsibilities. Partial responsibility should not directly translate into full legal liability, but into partial (proportionate) liability.^27^

It is important to note that the EU is already moving in this direction with the proposed AI Liability Directive (COM 2022). This directive, proposed by the European Commission in 2022 alongside the AI Act, introduces procedural rules regarding liability for AI technologies. It regulates liability claims that consumers may have for damage caused by AI-enabled products and services. In Article 4 of the directive, the burden of proof is reversed. With this, the directive creates a rebuttable ‘presumption of causality’ to ease the burden of proof for victims who want to establish any damage caused by AI systems.^28^

This suggested amendment of legal frameworks for liability law should be scoped toward complex technologies, not beyond this. For technologies that are less complex (e.g., not autonomous or self-learning), the current frameworks seem to function well. It is beyond the scope of this paper to look into liability law issues that do not involve technology. Any issues may not be comparable and most of the arguments provided here are much less likely to apply to other situations. But for complex technologies, this paradigm shift from liability gaps to liability overlaps may better reflect shared responsibilities. Based on some changes in legislation, courts and judges could further develop a body of case law on what distributions of responsibilities and liability (should) look like in different situations. This is not something new in tort law: in this field, most jurisdictions, both in civil law and common law systems, rely heavily on case law.

Shared responsibilities can help to better address liability gaps of type 3 (multiple actors) and type 4 (multiple legal regimes), as this approach could further develop fair and proportional distributions of liability. Shared responsibilities may also typically help address liability gaps of type 1 (in which no actor is liable), as with overlapping responsibilities, more actors come into scope. Note, however, that although this may decrease the probability that no actor is liable, this may still be the case, typically in highly autonomous systems. Hence, shared responsibilities will not solve all liability gaps of type 1. Likewise, shared responsibilities may not sufficiently address liability gaps of type 2 (the wrong actor is liable) in case the technology takes over significant parts of the decision-making. Even with shared responsibilities, it can be hard to hold the technology liable, as the technology itself is no legal entity. Although some members of the European Parliament suggested that AI and robots should perhaps have legal personhood in the future (Hern 2017; Daily Wire 2018), this would be very complicated and was rejected by the European Commission. Legal personhood for AI and robots would involve rights and duties for them, but it is unclear which rights and duties these would be and how that would work. In case of liability, the AI or robot would also need to have a budget to compensate any harm or damages and would need to have either sufficient conscience to make decisions or have a representative to make such decisions. Given these complications, another approach is suggested in this paper, which is extending responsibilities of actors through fiduciary duties (Sect. 3.3).

### Further extending responsibilities through fiduciary duties

A fiduciary duty is a concept mostly used in common law systems and describes the highest standard of care in law (Claassen 2024). A fiduciary is expected to behave beyond the standards of good faith and duty of care, and should be extremely loyal to the person to whom the duty is owned (the principal) (Williams 2021). Typically, the fiduciary is expected to ensure there is no conflict of duty between fiduciary and principal and must not profit from his position, unless the principal consents (Mantese 2020). In common law systems, fiduciary duties typically exist between an attorney and a client, a bank and a client, or a guardian and a ward. Important elements of a fiduciary duty include duty of care, loyalty, good faith, confidentiality, and prudence.

**Fig. 3:**
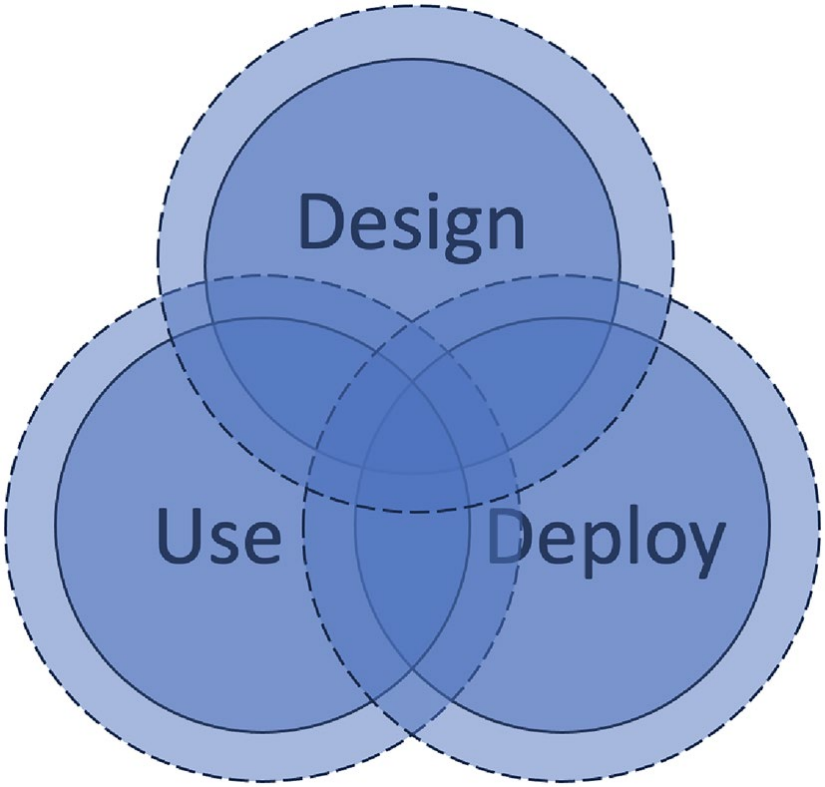
Potentially further extending responsibilities through fiduciary duties (dashed lines)

Although fiduciary duties are originally intended for relations in which one party takes care of the money, assets, legal position, or well-being of another person, this concept can also be useful for extending responsibilities regarding AI and other complex technologies. Fiduciary duties further extend responsibilities to a higher (or the highest) level of care in law. In other words, any existing responsibilities and liabilities are extended a bit further than they would exist under the liability mechanisms through fault and negligence, prevention, or benefits (Sect. 2.1). This is shown in Fig. 3, in which the dashed lines indicate fiduciary duties.

Introducing fiduciary duties would further increase the level of care that actors should take in developing, deploying, and using AI and other complex technologies (Cowger 2023). If the AI itself cannot be held responsible and liable because it has no legal personhood, then it should not be that no one is liable (type 1 responsibility gap) or that the nearest human actor is held liable (type 2 liability gap). Instead, based on fiduciary duties of all actors, the actors that have not shown sufficient levels of care should be held liable. In this way, fiduciary duties could help address type 1 and type 2 liability gaps.

Fiduciary duties can also play an important role in preventing any harm or damage from occurring (Benthall and Shekman 2023). The starting point of fiduciary duties is a high level of care. The focus on care, i.e., on high-quality technology that does not cause harm or damage, therefore is much more important than, for instance, profits. It brings more to the forefront a perspective of virtue ethics (in which intentions are central), rather than complying with the rules (like in deontology/duty ethics) (cf. Farina et al. 2022). Typically, the current focus on rules may not always be the best incentive to prevent developing, deploying or using technologies that can cause harm or damage. A focus on intentions and consequences could be more helpful in thinking about these technologies before they are developed, deployed, and used. From a legal perspective, fiduciary duties could further encourage this approach. In the EU, the AI Act seems to be heading in this direction. Adopted in 2024, the AI Act imposes high levels of care on the actors that develop, deploy, and use AI, particularly when this concerns types of AI that entail high(er) risk.

Given that fiduciary duties are a concept stemming from common law systems, it would require further research to investigate how they can be implemented and concretely applied in civil law systems. Transplanting legal concepts in this manner is difficult and does not always go as expected. When introducing fiduciary duties in their civil law legal frameworks, countries may change and adapt such a new concept to what is already available and looks similar in their legal system. Hence, there is the risk that an attempt to introduce fiduciary duties into the EU legal framework could end up being superseded by other tools that already exist. This is not necessarily problematic if the same or similar results are achieved. However, since this may lead to low levels of harmonization and may address liability gaps (much) less directly, there is the risk that this approach may not achieve its goals. Here, social contact theory—as opposed to social contract theory—could be helpful. Developed in Germany (Sozialen Kontakt, Dölle 1943) and Italy (contatto sociale, Santoro 2012) social contact theory is an example of human-centered design. It refers to contact or relationships between actors that is more than occasional and casual but does not amount to a full contract. Social contact can be a source of obligations under a duty of care, also in the context of responsibilities regarding AI and robotics (Da Conca 2020).

### Pros and cons

By acknowledging shared responsibilities and fiduciary duties in the process of designing, deploying, and using complex technologies such as AI, it is likely that liability gaps can be avoided or mitigated. Shared responsibilities may address liability gaps of type 1, type 3, and type 4. Fiduciary duties may address liability gaps of type 1 and type 2 in cases where the technology has high levels of automated decision-making. Shared responsibilities can be legally implemented in liability law by amending existing legislation, for instance, by assuming that all stakeholders are responsible and liable unless they can prove they have no control. Under international law, in the absence of a realistic prospect of a new multilateral treaty to this effect, customary international law, for example, in the field of international humanitarian law or human rights law, could gradually develop in this direction. Also, there should be more room for partial liability reflecting partial responsibilities, for instance, the responsibility of an actor to signal an issue without a corresponding responsibility to solve that issue. And, for any liability gaps caused by autonomous decision-making of AI, fiduciary duties could help find the right distribution of responsibility rather than having no one liable or liability of the nearest human operator. These approaches, already present to some extent in the AI Act and the procedural rules easing the burden of proof as proposed in the AI Liability Directive, can then be further detailed in case law, in which establishing and distributing liability can be assessed on a case-by-case basis, as finding the right distribution of responsibility may remain complicated in practice. Here the pros and cons of this approach from liability gaps to liability overlaps are examined. This begins by discussing three advantages of this approach and then discuss four disadvantages.

The first advantage is the most obvious: shared responsibilities and fiduciary duties directly address liability gaps and may create more satisfying results with regard to establishing and distributing responsibilities and legal liabilities. These will be better aligned. Typically, liability gaps may exist at the intersections of the different stages of putting complex technologies on the market (e.g., between design and deployment or between deployment and use). These parts of the technology life cycle are not always clearly connected to specific actors, and therefore liability gaps may occur. Recognizing shared responsibilities would much better reflect the fluid cooperation between actors in these stages, in which, for instance, developers and users co-create technology by providing input from different perspectives. Along with this involvement comes at least some level of control or influence that translates into responsibilities that, in turn, could be codified in legal liabilities. On top of this, fiduciary duties would help to take a much more preventive approach, in which the intentions and consequences are more central than compliance with the rules. This may contribute to preventing harm and damages caused by AI in the first place.

The second advantage is that shared responsibilities and fiduciary duties are likely to increase legal certainty. Liability gaps create legal uncertainty because they bring along a lack of liability or holding the wrong actor liable. Both are scenarios that do not contribute to trust among actors, particularly not among users who are currently at the end of a cascade where all residual liability ends. Legal certainty contributes to trust among users to use complex technologies as they can rest assured that if something goes wrong the right actor will be liable (which could be themselves, but even that is less problematic if morally right). It also contributes to trust among actors that design, develop, and deploy technologies. If users have more trust, they will have more incentives to invest in (developing) new technologies, as they have more clarity on the extent of their liabilities. The potential of shared responsibilities and fiduciary duties to reduce the number and extent of liability gaps may, therefore, increase legal certainty. Having said that, legal certainty may continue to exist to some extent, because finding the right distribution of responsibility may still be complicated in practice.

The third advantage is that shared responsibilities and fiduciary duties further facilitate cooperation between actors, improve understanding and, in the end, may lead to increased quality and safety of complex technologies. Shared responsibilities acknowledge that actors should (rather than could) involve other stakeholders. Typically, a manufacturer that does not involve users in the design and development of new technologies could be responsible and liable based on omission to do so, as this approach is likely to increase the risk for harm or damage. Together with fiduciary duties that encourage actors to consider intentions and outcomes of developing, deploying, and using AI technologies, shared responsibilities further encourage cooperation and co-creation among actors. This, in turn, will yield technologies that better take into account the different perspectives of various actors. The cooperation itself will yield a better understanding among actors and a better understanding of how the technology is designed and should be deployed and used, including less risk-taking behavior of actors. Altogether this could also result in the reduction of potential harm and damage. Genuine accidents will remain but, if they are handled well, these do not necessarily yield liability gaps.

The first disadvantage is that actors may see shared responsibilities and fiduciary duties as extensions of their (already burdensome) current liabilities. They may object to with this, as they may benefit from a limited scope of their liability. However, this may be a too narrow perspective: although limited liability is favorable when things go wrong, the bigger perspective may be that fewer things go wrong. Also, it should be considered that shared responsibilities sometimes extend liabilities, but may also involve shared or partial liabilities, which could mean a decreased (share in) liability.

The second disadvantage is that shared responsibilities and fiduciary duties may have a chilling effect on innovation. If companies that design and develop new technologies feel they are exposed to high liability risks, this may make them cautious and less risk-taking concerning innovation. Technology innovation often requires huge financial investments in research and development. Shared responsibilities and fiduciary duties may lead to incentives not to get involved in cooperation and co-creation of new technologies because the risks may be unclear. Avoidance of close cooperation could lead to reduced quality of the technology and more potential for harm and damages to occur. There could be an incentive to willingly avoid acquiring cognition of (the inner workings and risks of) particular technologies to avoid liability (Kwik 2023). The less people know, the more they will be able to deny any control or influence and, therefore, escape liability. This could be addressed by mandatory training programs, awareness campaigns, and clear policies on the deployment and use of technologies.

The third disadvantage is that in shared responsibilities (and to some extent also in fiduciary duties), there may be unclarity, disagreements, and disputes on how liability is shared and distributed. It could be argued that shared responsibilities simply move the problem from one place to another. However, this is not entirely correct. In the case of liability gaps, an actor confronted with harm or damage can try to find redress at each separate actor but may find all doors closed. In the case of shared responsibilities, all actors have to engage in a discussion on what happened and how much control and influence each actor had. Given a reversal of the burden of proof, they would have to actively show they had no control or influence whatsoever to escape liability. Therefore, although shared responsibilities and fiduciary duties do not directly offer a concrete solution, they open a pathway to collaboratively finding solutions. If actors are unable to resolve this together, courts and judges are enabled to further develop a body of case law for specific conditions and circumstances. Having said that, even though shared responsibilities and fiduciary duties will likely shift the focus to the burden of proof and to exculpating circumstances and exemptions to liability, it will remain complicated in practice to find the right distribution of responsibility.

The fourth disadvantage is that introducing shared responsibilities and fiduciary duties is unlikely to solve all liability gaps. Since AI and other complex technologies become more and more autonomous, they increasingly show emergent behavior that actors could not predict in any way. This inability to predict such behavior means the levels of control and influence of actors are limited, making it harder to distribute liability fairly, even when using concepts like shared responsibilities and fiduciary duties. The autonomy, complexity, opacity, and self-learning evolution of complex technologies like AI make it very difficult to assign liability. Often high levels of technological expertise are required to understand what is happening, and in some situations, it may even be technologically impossible to understand the inner workings of these technologies (e.g., black boxes) (Pasquale 2015). As a result, it may be difficult to determine how much control an actor has in the cooperation (Himmelreich 2019). Particularly there may be causality problems, i.e., problems isolating proper cause. These are not new problems. Already decades ago, the so-called problem of many hands was identified: due to a proliferation of actors to whom responsibility can be attributed, problems may arise in attributing responsibility (Nissenbaum 1996). Typically, this occurs in situations with multiple decision-makers in or across large organizations (Thompson 1980a). Shared responsibilities may not resolve this problem but may enable to look more detailed into the levels of control and influence of each of these actors.

## Conclusion

Liability gaps are situations in which applying existing liability rules yields morally unsatisfying results. In these situations, legal liabilities are not well-aligned with responsibilities. When dealing with AI and other complex technologies that may cause harm, such liability gaps may occur, typically when no actor can be held liable (type 1), when the wrong actor is held liable (type 2), when multiple actors are liable (type 3), or when there are multiple legal regimes (type 4). If the technology takes over decision-making, situations can emerge in which no actor may be liable. If the nearest human operator is held liable, this may be the wrong actor. In the case of multiple actors or legal regimes, actors may escape liability. In such cases, the solution may be morally unsatisfactory, as the harm and damage cannot be transferred to an actor who is responsible.

Responsibilities can stem from any control or influence that actors may have over the decision-making or (enabling/facilitating) the course of events leading up to any harm or damage. The traditional approaches in liability law (liability through fault or negligence, or forms of vicarious liability like strict liability or product liability) may not solve these liability gaps as they are based on models that look for putting liability fully on one or a few particular actors that are being sued or prosecuted. These actors are closely connected to the major stages of the process of putting complex technologies on the market, i.e., design, development, and manufacturing as the first stage, deployment as the second stage, and use as the third stage. This model typically functions as a cascade in which all residual liability ends at the user.

When disregarding the reality of legal liabilities and focusing more on actual practice and responsibilities, it can be observed that the development, deployment, and use of complex technologies are not distinguishable stages, as often suggested, but processes of cooperation and co-creation. The different stages may sometimes overlap or run in parallel, the cooperation can be fluid and control can be shared. The cooperation can lead to decisions and actions that cannot be traced to individuals or single entities (List and Pettit 2011; Björnsson 2020).

This paper, therefore, explored the concept of shared responsibilities. At the intersections of these stages, the shared responsibilities of multiple actors can be observed. Although none of the actors has complete control or a complete overview, many actors have some control or influence, and therefore responsibilities based on fault, prevention, or benefit. By focusing on shared responsibilities, liability gaps (particularly types 1, 3, and 4) can be turned into liability overlaps. Instead of no one being liable at the intersections of different stages or transferring liability to the wrong stage, actually, multiple actors are involved. Based on their level of control and influence, these actors can be held responsible. This responsibility, in turn, can be the grounds for creating legal liability based on fault, prevention, or benefit.

Shared responsibilities are unlikely to resolve liability gaps, particularly when the technology uses high levels of autonomy in decision-making. Since the technology itself cannot be held liable (as it is no legal entity), this paper proposes introducing fiduciary duties into the process of developing, deploying, and using AI. Fiduciary duties, setting the highest standard of care in law, may contribute to distributing responsibilities in fairer ways than simply holding no one liable (type 1) or blaming the most proximate human operator (type 2). Furthermore, fiduciary duties further emphasize the importance of intentions and outcomes of developing, deploying and using AI technology, rather than merely focusing on legal compliance. This aspect may contribute to preventing harm that AI and other complex technologies may cause.

In the EU, developments in the current legal frameworks for AI already reflect some of this, but would need some adjustment to reflect this. In the EU, the proposed AI Liability Directive introduces a rebuttable ‘presumption of causality’ to ease the burden of proof for victims who want to establish any damage caused by AI systems. The AI Act imposes high levels of care on the actors that develop, deploy, and use AI, particularly when this concerns types of AI that entail high(er) risk. Shared responsibilities could be further implemented in legislation by taking as a starting point the assumption that all stakeholders are responsible and liable unless they can prove they have no control. Customary international law could gradually develop in this direction, even if the prospect of a law-creating state practice to this effect does seem remote at this point. Also, any revised legislation should create more room for partial liability reflecting partial responsibilities. For instance, some actors may have a responsibility to signal or identify an issue whereas other actors may have a responsibility to solve that issue, depending on their respective levels of control and influence.

This approach from liability gaps to liability overlaps better aligns legal liabilities with responsibilities and has several advantages. It increases legal certainty, and increases cooperation and understanding between actors (in line with social contact theory), improving the quality and safety of technologies. Having said that, this may not solve all liability gaps, may have chilling effects on innovation, and may require further detailing through case law. Clearly, more research is needed on how to implement shared responsibilities and fiduciary duties in legal frameworks, particularly in civil law systems. Also, further research is needed on the complexities of finding the right distribution of responsibilities, which remains complicated even when shared responsibilities and fiduciary duties are used. As such, shared responsibilities and fiduciary duties are not a silver bullet to address liability gaps, but they may be a paradigm shift that better reflects the responsibilities of actors involved in complex technologies.

## Footnotes

1 The term ‘responsibility’ is used in this paper to refer to an actor’s duty, thus a relationship between the parties. It is used in a broader more general sense than legal responsibility, as distinguishable liability.

2 This is within the remits of *ultra vires*.

3 This could be under contract law or tort law.

4 This paper does not delve into the different moral frameworks that exist for establishing moral responsibilities. For defining responsibility gaps, it is sufficient here to consider that legal liabilities and moral responsibilities are not aligned (Matthias 2004; Danaher 2016).

5 This is closely related to virtue ethics (cf. Vallor 2016).

6 In this context, it is important to note that whereas civil law jurisdictions generally know a duty to act (e.g., Sect. 323c German Criminal Code: Failure to render assistance), common law jurisdictions generally only know a duty to not contribute to a deterioration of a situation.

7 In fact, some authors claim that it may even be irresponsible not to use certain technologies such as AI in military decision-support systems or in weapon systems (cf. Meerveld et al. 2023).

8 See, for instance, EU Directive 2011/53/EU (Consumer Rights Directive) and EU Regulation 2016/679 (General Data Protection Regulation).

9 Note that in the airline example, passengers can claim the airline under the Montreal Convention and the airline can then sue the true cause of the harm. However, in practice, this is rarely done as it is expensive and can unsettle complex and expensive contractual relationships.

10 Note that the AI Liability Directive proposed by the EU tries to address this by creating a rebuttable ‘presumption of causality’ to ease the burden of proof for victims to establish any damage cause by AI systems. See COM (2022).

11 In ethics, the discussion focuses on responsibility gaps rather than liability gaps (see Matthias 2004). Different types of responsibility gaps can be distinguished (see Santoni de Sio and Mecacci 2021).

12 https://www.uu.nl/en/organisation/in-depth/unboxing-the-black-box-of-ai

13 Autonomous operations are not the same as automatic operations. EASA has stated that an automatic operation is whereby the drone follows “pre-programmed instructions that the UAS executes while the remote pilot is able to intervene at any time.” Regulation 2019/947, GM1 Article 2(17)—Definitions—Definition of ‘Autonomous Operation’.

14 Some even deny the existences of responsibility gaps (see Tigard 2021).

15 Some even deny the existence of liability gaps altogether (see Wood 2023).

16 For example, if a person who has a legal custodian gets struck by lightning because the custodian failed to keep the person inside the house during a thunderstorm, the custodian could be held liable if a breach of duty of care is established.

17 Some refer to this as judicious liability gaps, indicating that such a liability gap is acceptable (cf. Kwik 2023).

18 Some suggest that if no one can be held responsible, deployment of such technology would be unethical (see Sparrow 2007).

19 Note that exactly for this reason, the EU AI Act focuses on putting AI technology on the market. Through such market controls, jurisdiction issues can be mitigated, although it is unlikely to prevent all problematic technologies from entering the EU markets.

20 The term life cycle should not be confused with product life cycles in which products are intruded in markets and decline after the end of their life cycle. When referring to the circularity of complex technology life cycles, this paper considers that their use involves a continuous learning curve with periodic reflections upon the original purposes of a given technology. Such reflection often includes changes, for instance, in prognostic criteria or even on more fundamental levels of technology design, to better meet new societal developments.

21 Note that the aircraft example addresses this. In 1955, the Hague Protocol stated that actual and contractual agents are to be both held liable. Servant and agents are also to be held liable under the airlines activities, leading to the airline being liable.

22 Here it is important to distinguish between safety critical technologies and non-safety critical technologies. Beta testing can be done for the latter, but may be complicated for the former. Hence, a high level of safety must be achieved for safety critical technologies before they are deployed. This impacts the user improvement feedback loop.

23 The technologies also may converge, as they build on each other (Custers et al. 2008).

24 A typical example of irresponsible users was the in situ training of Microsoft Chatbot Tay, that became racist in only 16 h (see Vincent 2016).

25 Others use the term collective responsibility (see Conradie 2023). The term shared responsibilities is used, relating to all actors involved at a certain stage of the process, whereas collective responsibilities might suggest all actors involved in the entire process. Collective responsibility is much more diffuse and harder to translate into legal liability (see Collins 2018).

26 Obviously, this assumes that there is a governance structure that facilitates this. Such governance structures for providing feedback or raising issues are often lacking in current practice.

27 Along with this, also procedural law should be addressed. If partial liability of several actors better reflects moral responsibilities that full liability of one actor, justice should not be impeded by very high transactional costs of suing ten actors rather than one single actor.

28 Note that at the time of writing (June 2024) the AI Liability Directive as still a proposal and not yet adopted by the EP and EC.
